# Association of Serum Malondialdehyde Levels with Lipid Profile and Liver Function in Patients with Inflammatory Bowel Disease

**DOI:** 10.3390/antiox13101171

**Published:** 2024-09-26

**Authors:** Nayra Merino de Paz, Marta Carrillo-Palau, Alejandro Hernández-Camba, Pedro Abreu-González, Antonia de Vera-González, Alejandra González-Delgado, Candelaria Martín-González, Miguel Á. González-Gay, Iván Ferraz-Amaro

**Affiliations:** 1 Division of Dermatology, Dermamedicin Clínicas, 38004 Santa Cruz de Tenerife, Spain; nayradepaz@hotmail.com; 2 Division of Gastroenterology, Hospital Universitario de Canarias, 38320 Tenerife, Spain; martacarry@yahoo.es; 3 Division of Gastroenterology, Hospital Universitario de Nuestra Señora de la Candelaria, 38010 Tenerife, Spain; dr.alejandrohc@gmail.com; 4 Unit of Physiology, Department of Basic Medical Sciences, University of La Laguna, 38200 Tenerife, Spain; pabreu@ull.edu.es; 5 Division of Central Laboratory, Hospital Universitario de Canarias, 38200 Tenerife, Spain; adeverag@gmail.com (A.d.V.-G.); alejandra.gd88@gmail.com (A.G.-D.); 6 Department of Internal Medicine, Universidad de La Laguna (ULL), 38200 Tenerife, Spain; mmartgon@ull.edu.es; 7 Division of Rheumatology, IIS-Fundación Jiménez Díaz, 28040 Madrid, Spain; 8 Department of Medicine and Psychiatry, University of Cantabria, 39011 Santander, Spain; 9 Division of Rheumatology, Hospital Universitario de Canarias, 38320 Tenerife, Spain

**Keywords:** ulcerative colitis, Crohn’s disease, inflammatory bowel disease, malondialdehyde serum levels, oxidative stress

## Abstract

Malondialdehyde (MDA) is a naturally occurring organic compound produced as a byproduct of lipid peroxidation. It serves as one of the most widely recognized biomarkers for oxidative stress. Elevated levels of MDA have been observed in patients with inflammatory bowel disease (IBD), suggesting its involvement in the pathogenesis and progression of the disease. In this study, we analyzed MDA levels within a well-characterized and extensive cohort of IBD patients. Our objective was to investigate the association between MDA levels and disease characteristics in this population. This is a cross-sectional study that encompassed 197 patients with IBD. Multivariable linear regression analysis was performed to study the relationship between disease characteristics and circulating MDA. MDA was significantly associated with male sex in IBD patients but not with other demographic characteristics or classic cardiovascular risk factors. Regarding disease features such as phenotype or activity indices, their relationship with MDA was scarce. Several lipid profile molecules showed a significant association with MDA levels after multivariable analysis. Similarly, the liver fibrosis-4 index and hepatic elastography values were significantly related to higher MDA levels after adjusting for covariates. In conclusion, the sources of elevated MDA in IBD are primarily linked to lipid profile abnormalities and liver disease.

## 1. Introduction

Inflammatory bowel disease (IBD) comprises two main disorders: ulcerative colitis (UC) and Crohn’s disease (CD). While UC affects the colon, CD can affect any component of the gastrointestinal tract from the mouth to the perianal area. Both are considered inflammatory conditions characterized by relapsing and remitting episodes. Inflammation in UC is limited to the mucosal layer of the colon whereas CD is characterized by transmural inflammation and by skip areas of involvement. Pathogenesis is thought to be multifactorial, involving genetic predisposition, immune dysregulation, environment, and microbiome [1]. Extraintestinal conditions associated with IBD may be present at diagnosis or develop later in the disease course. These may involve the skin, joints, hepatobiliary tract, eye, kidney, and rarely pancreas and respiratory systems, as well as risks for venous thrombosis [2]. Other extraintestinal and increasingly prevalent disorders with significant complications and impacts on future health burden in IBD include an increased risk of atherosclerotic cardiovascular disease [3,4] and the co-existence of metabolic dysfunction-associated steatotic liver disease (MASLD), previously known as nonalcoholic fatty liver disease [5].

Oxidative stress is a phenomenon caused by an imbalance between production and accumulation of oxygen reactive species (ROS) in cells and tissues and the ability of a biological system to detoxify these reactive products. ROS play essential physiological roles, being naturally produced as by-products of oxygen metabolism [6]. For instance, they are key in redox signaling, which is vital for cellular processes such as growth and differentiation. In immune responses, phagocytic cells like neutrophils and macrophages generate ROS to eliminate invading pathogens. Additionally, ROS are involved in regulating vascular tone [6]. Besides, lipid peroxidation or the reaction of oxygen with unsaturated lipids produces a wide variety of oxidation products. The main primary products of lipid peroxidation are lipid hydroperoxides. Among the many different aldehydes which can be formed as secondary products during lipid peroxidation, malondialdehyde (MDA) has been widely used for many years as a convenient biomarker for lipid peroxidation because of its facile reaction with thiobarbituric acid [7]. Elevated MDA levels have been associated with a wide range of diseases, including cardiovascular diseases, diabetes, neurodegenerative disorders, inflammatory diseases and cancers [8,9]. Besides, MDA levels are often measured to assess the effectiveness of antioxidant therapies. A decrease in MDA levels after treatment can indicate that the therapy is successful in reducing oxidative stress.

Among other immunoregulatory factors, ROS are produced at abnormally high levels in IBD. This includes MDA, which has been shown to be elevated in patients with IBD, with its levels correlating with disease severity [10]. However, the mechanisms driving the increase in MDA levels in IBD remain unknown, as do the specific disease characteristics responsible for this elevation. In this context, most previous studies have generally included a limited number of patients and have not provided a comprehensive characterization that includes extraintestinal manifestations, cardiovascular or liver disease, a complete lipid profile, or insulin resistance indices.

In this study, we measured MDA levels in a well-characterized and extensive cohort of patients with IBD. This characterization included a comprehensive lipid profile, the assessment of subclinical carotid atherosclerosis, and liver disease evaluation through elastography and ultrasound. We then performed a multivariable analysis to investigate the associations between MDA levels and various disease characteristics

## 2. Materials and Methods

### 2.1. Study Participants

A cross-sectional study was conducted involving 197 consecutive patients diagnosed with IBD, all of whom were 18 years of age or older. These individuals were under the care of gastroenterologists and received regular follow-ups at gastroenterology outpatient clinics. Inclusion criteria were diagnosis of IBD based on clinical, endoscopic, and histological criteria. To be included in this study, patients with IBD were required to have a disease duration of ≥1 year. Exclusion criteria for all participants included a history of cancer, any other chronic inflammatory or autoimmune disease, or evidence of active infection. Additionally, patients with autoimmune hepatitis, alcoholism, or hepatitis virus infection were excluded. The study protocol was approved by the Institutional Review Committees at Hospital Universitario de Canarias and Hospital Universitario Nuestra Señora de La Candelaria, both located in Spain, and all participants provided written informed consent (approval no. CHUC_2019_103). Research involving human subjects adhered to the principles of the Helsinki Declaration.

### 2.2. Data Collection

Questionnaires regarding clinical history were conducted in both IBD patients and control groups to evaluate cardiovascular risk factors and medication usage. Hypertension was defined as a systolic blood pressure exceeding 140 mmHg or a diastolic blood pressure exceeding 90 mmHg. Disease activity in patients with CD was determined using two measures: the Crohn’s Disease Activity Index (CDAI) and the Harvey-Bradshaw Index (HBI) [11]. CDAI was categorized as 0 to 149: Asymptomatic remission; 150 to 220 points: Mildly to moderately active; 221 to 450 points: Moderately to severely active; 451 to 1100 points: Severely active to fulminant disease. HBI was categorized as 0 to 4 points Clinical remission; 5 to 7 points: Mildly active disease; 8 to 16 points: Moderately active disease; 17 to 100 points: Severely active disease. Disease activity in UC was calculated through the partial Mayo Clinic score [12]. Dyslipidemia was defined as meeting one or more of the following criteria: total cholesterol exceeding 200 mg/dL, triglyceride levels exceeding 150 mg/dL, HDL-cholesterol lower than 40 mg/dL in men or less than 50 mg/dL in women, or LDL-cholesterol surpassing 130 mg/dL. Information regarding the therapies used in the disease was collected including the use of mesalazine, prednisone (as binary or mg/day), azathioprine and methotrexate, and biological therapies.

The Systematic Coronary Risk Evaluation-2 (SCORE2) cardiovascular risk tool was calculated as previously described using age, gender, smoking status, systolic blood pressure, and non-HDL-cholesterol [13]. SCORE2 estimates an individual’s 10-year risk of fatal and non-fatal CV disease events in individuals aged 40 to 69 years. For healthy people aged ≥70 years, the SCORE2-OP (older persons) algorithm estimates 5-year and 10-year fatal and nonfatal CV disease events.

A carotid ultrasound examination was conducted to assess the thickness of the carotid intima-media wall (cIMT) in the common carotid artery and to detect any localized plaques in the extracranial carotid arteries. Measurements were performed using the Esaote MyLab 70 ultrasound system from Genova, Italy, which features a 7–12 MHz linear transducer and utilizes the Quality Intima Media Thickness (QIMT) automated software-guided radiofrequency technique version 6 developed by Esaote in Maastricht, The Netherlands. The assessment followed the Mannheim consensus guidelines [14], which outline criteria for identifying plaques in the accessible extracranial carotid arteries, including the common carotid artery, the bulb, and the internal carotid artery. Plaque was defined as a localized bulge within the arterial lumen with a cIMT measurement greater than 1.5 mm, where the bulge was at least 50% larger than the adjacent cIMT or resulted in a reduction in the arterial lumen by more than 0.5 mm [14].

### 2.3. MDA Assessment

The Thiobarbituric Acid Reactive Substance (TBARS) assay is a method employed to detect lipid oxidation. This assay specifically measures MDA, one of the end products generated during the breakdown of lipid peroxidation compounds. Serum levels of MDA were determined using a modified version of the method described by Kikugaw et al. [15]. To perform the assay, a 0.2 mL volume of the sample was combined with 0.2 mL of 0.2 M H_3_PO_4_ (purity 85%, Merck Life Science, Madrid, Spain). The color reaction was initiated by adding 25 µL of a 0.11 M thiobarbituric acid (TBA, purity 100%, Sigma-Aldrich, Madrid, Spain) solution. The mixture was then heated at 90 °C for 50 min using a heating block. After cooling, the TBARS (resulting in a pink complex color) were extracted by adding 0.4 mL of n-butanol (purity 100%, Sigma-Aldrich, Madrid, Spain). Centrifugation at 6000× *g* for 10 min was performed to separate the butanolic phase. Each sample was transferred to a 96-well plate and read at 535 nm using a microplate spectrophotometer reader (Spectra MAX-190, Molecular Devices, Sunnyvale, CA, USA). A calibration curve was prepared using authentic MDA standards (purity 100%, Merck Life Science, Madrid, Spain). The detection limit of the assay was established at 0.079 nmol/mL. The intra- and inter-assay coefficients of variation were calculated as 1.82% and 4.01%, respectively. The serum concentration of MDA was expressed in nmol per mL. To minimize potential interferences from compounds that react or absorb at 532 nm, each sample was accompanied by a blank tube (sample without the TBA reagent). The absorbance of the blank tube was subtracted from the absorbance measurements of each sample [16]. Additionally, the use of butanol as the extracting agent for the TBARS complex helped to mitigate many of these interferences [17].

### 2.4. Laboratory Assessments

The erythrocyte sedimentation rate (ESR) was determined using the Westergren method. High-sensitivity C-reactive protein (hs-CRP) levels were measured using a high-sensitivity immunoassay. Cholesterol, triglycerides, and HDL cholesterol were measured using the enzymatic colorimetric assay (Roche, Barcelona, Spain). Lipoproteins were assessed using a quantitative immunoturbidimetric assay (Roche, Barcelona, Spain). Cholesterol ranged from 0.08 to 20.7 mmol/L (intra-assay coefficient of variation of 0.3%); triglycerides ranged from 4 to 1.000 mg/dL (intra-assay coefficient of variation of 1.8%); and HDL cholesterol ranged from 3 to 120 mg/dL (intra-assay coefficient of variation of 0.9%). The atherogenic index was calculated using the total cholesterol: HDL cholesterol ratio according to the Castelli formula. LDL cholesterol was calculated using the Friedewald formula.

The homeostatic model assessment (HOMA) method was performed to determine IR. Briefly, the HOMA model enabled an estimation of insulin sensitivity (%S) and β-cell function (%B) based on fasting plasma insulin, C peptide, and glucose concentrations. In this study, we used HOMA2, the updated computer-based HOMA model [18]. This model can be used to assess insulin sensitivity and beta cell function from paired fasting plasma glucose and specific insulin, or C peptide, concentrations over a range of 1–2200 pmol/L for insulin and 1–25 mmol/L for glucose. C-peptide provides a better estimate of β-cell function as it is a direct marker of insulin secretion. Additionally, insulin data are preferable for calculating %S, as HOMA-%S is derived from glucose disposal in relation to insulin concentration. In our study, IR and %S were calculated using insulin serum levels. Otherwise, %B was calculated using C-peptide serum levels. The computer model provided a value for insulin sensitivity expressed as HOMA2-%S (in which 100% is normal). HOMA2-IR (insulin resistance index) is simply the reciprocal of %S.

### 2.5. Liver Disease Assessments

Abdominal ultrasonography in B mode was performed on patients with IBD to assess the degree of steatosis based on the extent of fat infiltration. In this regard, fat infiltration was classified into three degrees as previously described [19,20]: mild, when there was a discrete diffuse increase in hepatic echogenicity, clearly displaying the diaphragmatic line and intrahepatic vascular structures; moderate, when intermediately hepatic echogenicity was observed compared to that of the kidney, as well as mean attenuation of the diaphragmatic wall and intrahepatic vessels and; severe, when there was a significant difference in hepatic and renal echogenicity, an absence of diaphragm visualization and attenuation of vessels without being able to visualize them at the hepatic posterior pole. Transition elastography or Fibroscan^®^ was used to noninvasively establish the degree of hepatic fibrosis. Ten valid measurements were made, with a success rate of 60% or greater and an interquartile range of less than 30%, to determine the validity of the results. The degree of hepatic fibrosis was established according to F0 (no fibrosis) to F4 (cirrhosis) stages. Fibroscan^®^ values correlated with liver fibrosis as follows: <7.6 KPa = F0–F1, 7.7–9.4 KPa = F2, 9.5–14 KPa = F3, >14 KPa = F4 [21,22]. Both abdominal ultrasound and elastography procedures were performed after 6 h of fasting.

FIB-4 (Fibrosis-4) is a non-invasive scoring system used to estimate liver fibrosis or cirrhosis. It uses a formula that incorporates age, platelet count, and levels of alanine aminotransferase (ALT) and aspartate aminotransferase (AST). FIB-4 was calculated using the equation: FIB-4 = Age × AST/(0.001 × Platelets × square root (ALT) [23]. Cut-offs used are low-risk for fibrosis < 1.45 point, indeterminate-risk ≥ 1.45 and ≤3.25 points, and high-risk > 3.25 points.

### 2.6. Statistical Analysis

Demographic and clinical characteristics were presented as frequencies for binary variables. Continuous variable data were expressed as either mean ± standard deviation (SD) or as a median and interquartile range (IQR) for variables that did not follow a normal distribution. Disease-related data in relation to MDA was assessed using multivariable linear regression analysis. Confounding variables were selected from demographic factors and traditional cardiovascular risk factors if their *p*-values were less than 0.20 in the univariate analysis comparing patients and controls. All statistical analyses were carried out utilizing Stata software, version 17/SE (StataCorp, College Station, TX, USA), and a significance level of 5% was adopted for two-sided tests. A *p* value less than 0.05 was considered indicative of statistical significance.

## 3. Results

### 3.1. Demographics and Disease-Related Data

A total of 197 IBD patients with a mean ± SD age of 49 ± 10, respectively, were included in this study. Demographic and disease-related characteristics of the participants are detailed in Table 1. The BMI was 27 ± 5 kg/m^2^, with 28% of patients being obese (BMI ≥ 30). Regarding cardiovascular risk factors, 20% were smokers, 6% were diabetic, and 18% were hypertensive. Additionally, 4% of patients were on aspirin, and 11% were on statins.

Among the IBD patients, 66% had CD and 34% had UC. The median disease duration for IBD was 12 years (IQR 8–19). In patients with CD, the predominant phenotypes were ileal and non-stricturing, non-penetrating. The median CDAI score was 39 (IQR 7–80), and 89% of the patients were classified as being in asymptomatic remission. Similarly, the HBI had a median score of 2 (IQR 0–4), with 82% of patients in the remission category based on this index. For UC, 52% had experienced pancolitis, and 78% had a partial Mayo score of less than 2 points. Further details concerning disease-related data can be found in Table 1.

### 3.2. Inflammatory Bowel Disease Data Relation to MDA Serum Levels

The relationship between demographic characteristics and serum MDA levels is shown in Table 2. Being male was associated with significantly higher MDA levels compared to females. In contrast, body composition values and cardiovascular risk factors did not show a significant relationship with MDA levels.

Regarding disease characteristics, some significant relationships were observed after covariable adjustment for those demographic variables that showed an association with a *p*-value below 0.20 in the univariable analysis (sex, BMI, hypertension, and dyslipidemia). Specifically, while CRP values showed a significant relationship with lower MDA levels, Crohn’s disease types A2 and L2 exhibited higher MDA levels compared to other phenotypes. Remarkably, disease activity scores of CD and UC did not exhibit significant associations with MDA levels. Fecal calprotectin levels also did not disclose a significant association with MDA values. In terms of disease treatments, the use of mesalazine was associated with significantly lower MDA levels, whereas the use of anti-tumor necrosis factor therapies was related to significantly higher MDA values (Table 2).

### 3.3. Cardiovascular Disease Data Relation to MDA Serum Levels

**Figure 1:**
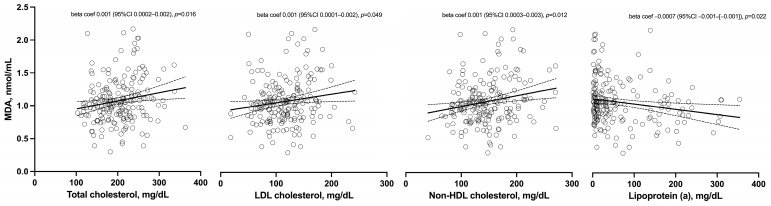
Relation of total, LDL, non-HDL cholesterols, and lipoprotein (a) to MDA values. Bold lines depict the linear regression, while dashed lines represent the 95% confidence interval. Circles represent individual values for each subject. Beta coef: beta coefficient; CI: Confidence interval.

Associations between cardiovascular-related data and MDA are shown in Table 3 and Figure 1. The SCORE2 calculator, whether considered continuously or categorically, did not show a significant association with MDA levels. Similarly, glucose homeostasis molecules and insulin resistance indices were not related to MDA. Moreover, cIMT and the presence of plaque (unilateral or bilateral) did not show a significant relationship with MDA. Finally, many lipid profile molecules showed significant associations with MDA. In this regard, after adjusting for covariates, levels of cholesterol, LDL, and non-HDL cholesterol were associated with higher MDA values. In the case of lipoprotein (a), this relationship was also significant but negative (Table 3 and Figure 1).

### 3.4. Liver Disease Parameters Association with MDA Values

Values of FIB-4, liver stiffness using Fibroscan^®^, and grade of steatosis through ultrasound are shown in Table 4. In patients with IBD, the mean FIB-4 score was 0.93 ± 0.42. Upon categorization, 89% of patients were classified as low risk for fibrosis, while 11% fell into the indeterminate risk category based on this index. Notably, no patient had a FIB-4 score suggestive of high-risk fibrosis.

**Figure 2:**
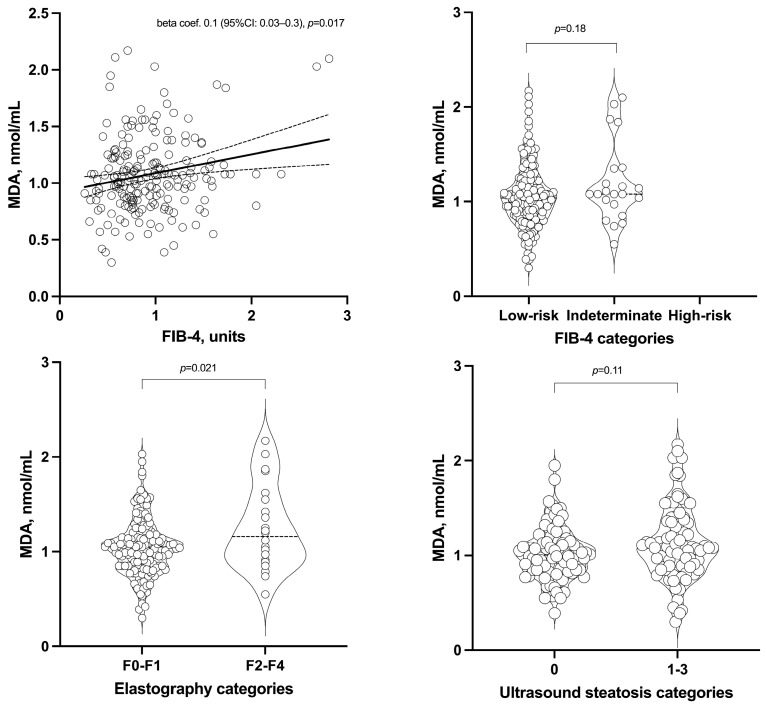
Relation of FIB-4 index as continuous and categorized, elastography categories and ultrasound steatosis categories to malondialdehyde (MDA) serum levels. Bold lines depict the linear regression, while dashed lines represent the 95% confidence interval. Circles represent individual values for each subject. In the violin plots median is shown. Beta coef: beta coefficient; CI: Confidence interval.

Fibroscan assessment of liver fibrosis showed a mean value of 0.97 ± 0.64 kPa. When these results were categorized, 86% of patients had stiffness values indicative of no or mild fibrosis (F0–F1), whereas 9%, 3%, and 2% had values corresponding to significant fibrosis, severe fibrosis, and cirrhosis, respectively. Additionally, ultrasound findings revealed that the majority of IBD patients (51%) had no hepatic steatosis, while 31% and 18% presented with mild and moderate or severe steatosis, respectively (Table 4 and Figure 2).

FIB-4 was significantly associated with higher circulating MDA levels even after multivariable analysis (beta coefficient 0.1, 95% CI: 0.03-0.3 nmol/mL, *p* = 0.017). Similarly, liver stiffness was initially associated with elevated MDA levels, but this significance was lost after multivariable adjustment. However, when liver stiffness was categorized (F2 to F4 versus F0), patients in the F2 to F4 categories showed significantly higher MDA levels after adjustment for covariates (beta coefficient 0.2, 95% CI: 0.03-0.3, *p* = 0.021). Likewise, patients with mild to severe steatosis had significantly higher MDA levels compared to those without steatosis in the univariable analysis. After adjustment, the significance was lost, though a trend persisted (Table 4 and Figure 2).

## 4. Discussion

Our study includes the largest cohort of patients in which MDA levels have been analyzed. In this regard, no previous study has provided as comprehensive a characterization of an IBD patient cohort as ours, which includes measurements of subclinical atherosclerosis, a complete lipid profile, insulin resistance indices, and assessments of hepatic disease. Our findings reveal a weak correlation between circulating MDA and disease characteristics. However, there is a significant association with lipid profiles and hepatic dysfunction, highlighting underlying aspects of the disease.

Levels of MDA have been analyzed in patients with IBD before, but these studies have been conducted in small cohorts and without performing multivariable analysis. In a study involving 24 patients with CD, 18 patients with UC, and 38 matched healthy subjects, plasma MDA levels were significantly higher in CD patients compared to the control group. However, MDA levels in CD patients were not significantly higher than those in UC patients [24]. In another report, MDA levels were analyzed in 30 patients with UC and 30 controls. It was found that MDA levels were significantly higher in the UC group compared to the control group [25]. However, there was no correlation between serum MDA level and disease activity. This was not observed in a study where 24 patients with active CD exhibited significantly higher serum levels of MDA compared to 25 patients with inactive disease. [26]. MDA has also been evaluated in intestinal biopsies of 17 patients affected by IBD (12 CD and 5 UC) and 12 healthy control individuals. MDA levels were significantly increased in biopsies from both UC and CD patients compared to biopsies from healthy controls [27]. Notably, these studies lacked multivariable analysis and were performed on a limited number of participants. Our study addresses these limitations by employing a robust methodology. We recruited a substantial cohort of patients, which provided sufficient statistical power to conduct a thorough multivariable analysis. This approach allows for the control of potential confounding factors and provides a more detailed understanding of the relationship between MDA levels and IBD.

In our study, while males exhibited higher MDA levels compared to females, demographic, body composition, and cardiovascular comorbidity variables were not significantly associated with MDA. Notably, disease activity indices for both CD and UC were also not associated with MDA levels. Similarly, while certain disease phenotypes showed associations with MDA, these associations were inconsistent, and it cannot be concluded that a particular disease pattern is related to higher MDA values. Besides, after multivariable adjustment, patients using mesalazine exhibited lower MDA values, which could suggest a potential antioxidant effect of this medication. Conversely, patients on anti-tumor necrosis factor therapy disclosed higher MDA values. This potentially indicates a link between MDA and disease activity, as these therapies are typically used for more severe cases. To our knowledge, no previous studies have evaluated the effect of anti-tumor necrosis factor agents on MDA levels. However, a prior study demonstrated that infliximab, an anti-TNF drug, reduces serum levels of conjugated dienes in patients with CD, suggesting an antioxidant effect of this medication [28]. However, the cross-sectional nature of our study precludes a definitive conclusion regarding the relationship between these therapies and MDA. Notably, no significant association was found between fecal calprotectin levels and MDA, either.

Remarkably, we found an independent and positive relationship between total and LDL-cholesterol and MDA in patients with IBD. Increased LDL-cholesterol levels are a risk factor for coronary diseases and its oxidized forms are especially atherogenic. In this regard, plasma levels of oxidized and MDA-modified LDL are associated with coronary artery disease [29]. While we did not specifically measure oxidized forms of the lipid profile in our study, MDA is a final product of oxidized LDL. Therefore, in IBD, there may be an atherogenic lipid profile, likely oxidized, which increases MDA levels. Consequently, elevated MDA values could also provide information about a deleterious atherogenic lipid profile and aid in making specific therapeutic decisions in IBD. However, in our study, we observed a negative relationship between lipoprotein (a) and MDA. This finding could have several explanations. In this regard, lipoprotein (a) is thought to have protective roles in vascular biology, such as promoting clot stabilization and tissue repair [30]. It might mitigate oxidative stress by binding oxidized lipids or having anti-inflammatory effects, thereby potentially reducing MDA levels. On the other hand, if lipoprotein (a) is elevated, it could be part of a compensatory mechanism that modulates oxidative stress or inflammation, leading to lower MDA levels. This compensation might be due to lipoprotein (a)’s role in trapping and neutralizing oxidized lipids. This negative relationship could also reflect complex interactions between lipoprotein (a) and oxidative processes that are not fully understood. For instance, lipoprotein (a) might interact with various pathways that influence oxidative stress in ways that do not directly correlate with MDA levels.

We did not find an association between circulating MDA and insulin resistance in IBD patients. The relationship between IBD and insulin resistance remains controversial. Some studies have reported that IBD patients in remission have normal glucose metabolism and insulin resistance similar to healthy controls, while others suggest that inflammation plays a role in insulin resistance in these patients [31]. Likewise, oxidative stress has been implicated in the pathogenesis of insulin resistance in healthy populations [32]. According to our findings, there is no association between MDA and insulin resistance in IBD patients.

Liver involvement may occur in IBD [33]. The most well-documented association in this context is between IBD and primary sclerosing cholangitis. Furthermore, drug-induced hepatotoxicity represents a significant cause of liver disease in IBD patients. Moreover, the prevalence of MASLD has been shown to be higher in IBD patients compared to the general population [34]. Additionally, patients with MASLD have been reported to have elevated levels of lipid peroxidation end-products, including circulating MDA [35]. In our study, we observed a consistent positive relationship between liver disease—measured using the FIB-4 index, elastography, or ultrasound—and MDA levels. We believe that this relationship, which is evident in the general population, is likely maintained in patients with IBD.

We acknowledge the limitation of not including control subjects in our study. However, this was not the aim, as differences in MDA levels between IBD patients and controls have been addressed in previous research. Additionally, the cross-sectional nature of our study prevents us from inferring causality. Finally, some data that was not recorded in our study like dietary intake, as well as other lifestyle parameters, could have influenced MDA values assessments.

## 5. Conclusions

In conclusion, the source of MDA expression in patients with IBD stems from a complex interplay of factors that primarily involves their lipid profile and the liver disease accompanying the condition. This relationship highlights the intricate connection between oxidative stress, lipid metabolism, and the inflammatory processes characteristic of IBD. Remarkably, this finding persisted despite the absence of a significant relationship between MDA levels and specific disease characteristics such as phenotypes and clinical activity.

Our study opens new avenues for research including the need for longitudinal studies to track changes in MDA levels over time, correlating these changes with disease progression and treatment responses. Additionally, it highlights the importance of delving deeper into the specific pathways and mechanisms by which lipid metabolism and liver dysfunction contribute to increased MDA production in IBD patients. Furthermore, exploring the potential use of antioxidant drugs as a therapeutic approach to this disease could provide valuable insights into the complex relationships between oxidative stress, lipid metabolism, and IBD pathogenesis, ultimately leading to improved diagnostic and therapeutic strategies.

## Figures and Tables

**Table 1:** Characteristics of patients with inflammatory bowel disease.

	IBD Patients
	(n = 197)
Malondialdehyde, nmol/L	1.08 ± 0.30
Age, years	49 ± 10
Male, n (%)	107 (54)
Body mass index, kg/m^2^	27 ± 5
Waist circumference, cm	94 ± 12
Hip circumference, cm	101 ± 11
Waist to hip ratio	0.93 ± 0.07
Cardiovascular co-morbidity	
Smoking, n (%)	39 (20)
Diabetes, n (%)	11 (6)
Hypertension, n (%)	35 (18)
Dyslipidemia, n (%)	155 (79)
Obesity, n (%)	55 (28)
Aspirin, n (%)	7 (4)
Statins, n (%)	21 (11)
IBD related data	
Crohn’s disease, n (%)	130 (66)
Ulcerative colitis, n (%)	67 (34)
CRP, mg/L	1.8 (0.9–3.8)
Disease duration since diagnosis, years	12 (8–19)
Crohn’s Disease related data, n (%)	
A1 below 16 years	19 (15)
A2 between 17 and 40 years	81 (62)
A3 above 40 years	27 (21)
L1 ileal	56 (43)
L2 colonic	23 (18)
L3 ileocolonic	51 (39)
L4 isolated upper disease	11 (8)
B1 non-stricturing, non-penetrating	73 (56)
B2 stricturing	46 (35)
B3 penetrating	14 (11)
CDAI score	39 (7–80)
Asymptomatic remission	116 (89)
Mild to moderate active Crohn disease	10 (8)
Moderate to severe active Crohn disease	3 (2)
Severely active to fulminant disease	0 (0)
Harvey-Bradshaw Index	2 (0–4)
Clinical remission	106 (82)
Mildly active disease	14 (11)
Moderately active disease	8 (6)
Severely active disease	1 (1)
Ulcerative Colitis related data, n (%)	
Proctosigmoiditis	7 (10)
Left-sided colitis	23 (35)
Pancolitis	34 (52)
Partial Mayo score	1 (0–1)
<2	52 (78)
>=2	15 (21)
Fecal calprotectin, mcg/g	113 (30–251)
>150	96 (49)
>=150	71 (36)
Perianal disease, n (%)	23 (12)
Previous surgery, n (%)	55 (28)
Current prednisone, n (%)	6 (2)
Prednisone, mg/day	8 (5–20)
Oral mesalazine, n (%)	175 (89)
Methotrexate, n (%)	22 (11)
Azathioprine, n (%)	61 (31)
Anti-TNF therapy, n (%)	58 (29)
Ustekinumab, n (%)	8 (4)
Vedolizumab, n (%)	5 (3)
Tofacitinib, n (%)	4 (2)

Data represent means ± SD or median (interquartile range) when data were not normally distributed. CRP: C reactive protein; TNF: tumor necrosis factor; CDAI: Crohn’s Disease Activity Index.

**Table 2:** Inflammatory bowel disease data relation to MDA serum levels.

	MDA, nmol/mL			
	Beta Coefficient (95% Confidence Interval), *p*			
	Univariable		Multivariable	
Age, years	0.0004 (−0.004–0.005)	0.86		
Male	**0.1 (0.02–0.2)**	**0.017**		
Body mass index, kg/m^2^	0.007 (−0.002–0.01)	0.14		
Waist circumference, cm	0.003 (−0.001–0.007)	0.17		
Hip circumference, cm	0.003 (−0.002–0.007)	0.27		
Waist to hip ratio	0.2 (−0.5–0.9)	0.51		
Cardiovascular co-morbidity				
Smoking	0.03 (−0.09–0.2)	0.59		
Diabetes	−0.03 (−0.2–0.2)	0.81		
Hypertension	0.09 (−0.03–0.2)	0.14		
Dyslipidemia	0.08 (−0.04–0.2)	0.17		
Obesity	0.03 (−0.08–0.1)	0.64		
Aspirin	0.03 (−0.08–0.1)	0.64		
Statins	−0.09 (−0.2–0.06)	0.24		
IBD related data				
Crohn’s disease	-			
Ulcerative colitis	−0.02 (−0.1–0.09)	0.76		
CRP, mg/L	**−0.02 (−0.03–(−0.01))**	**<0.001**	**−0.02 (−0.03–(−0.01))**	**<0.001**
Disease duration, years	−0.005 (−0.01–0.0004)	0.073	−0.004 (−0.009–0.001)	0.11
Crohn’s Disease data				
A1 below 16 years	−0.1 (−0.3–0.03)	0.11		
A2 between 17 and 40 years	0.09 (−0.02–0.2)	0.098	**0.1 (0.02–0.2)**	**0.022**
A3 above 40 years	−0.06 (−0.2–0.07)	0.37		
L1 ileal	−0.03 (−0.1–0.09)	0.64		
L2 colonic	**0.2 (−0.04–0.3)**	**0.013**	**0.2 (−0.006–0.3)**	**0.042**
L3 ileocolonic	−0.1 (−0.2–0.02)	0.097	−0.1 (−0.2–(−0.00004))	0.050
L4 isolated upper disease	0.01 (−0.2–−0.2)	0.92		
B1 non-stricturing, non-penetrating	−0.03 (−0.1–0.08)	0.65		
B2 stricturing	0.08 (−0.04–0.2)	0.18	0.09 (−0.02–0.2)	0.14
B3 penetrating	−0.01 (−0.2–0.2)	0.87		
CDAI score	−0.0007 (−0.001–0.00007)	0.074	−0.0004 (−0.001–0.0004)	0.34
Asymptomatic remission	-			
Mild to moderate active	−0.07 (−0.3–0.1)	0.50		
Moderate to severely active	0.1 (−0.3–0.5)	0.52		
Severely active to fulminant	-			
Harvey-Bradshaw Index	−0.008 (−0.03–0.01)	0.39		
Clinical remission	-			
Mildly active disease	0.05 (−0.1–0.2)	0.60		
Moderately active disease	−0.1 (−0.4–0.1)	0.32		
Severely active disease	−0.1 (−0.8–0.5)	0.72		
Ulcerative Colitis related data, n (%)				
Proctosigmoiditis				
Left-sided colitis	0.2 (−0.04–0.3)	0.12	0.2 (−0.02–0.4)	0.086
Pancolitis	−0.03 (−0.2–0.2)	0.75		
Partial Mayo score	−0.04 (−0.1–0.02)	0.14	−0.05 (−0.1–0.01)	0.11
<2	-			
>=2	−0.2 (−0.4–0.03)	0.098	−0.2 (−0.4–0.02)	0.071
Fecal calprotectin, mcg/g	0.00006 (−0.00006–0.0002)	0.32		
>150	-			
>=150	−0.04 (−0.1–0.05)	0.40		
Perianal disease	−0.07 (−0.2–0.08)	0.37		
Previous surgery	0.04 (−0.07–0.1)	0.50		
Current prednisone	−0.02 (−0.3–0.3)	0.91		
Prednisone, mg/day	0.009 (−0.01–0.03)	0.21		
Oral mesalazine	**−0.1 (−0.02–(−0.001))**	**0.048**	**−0.1 (−0.2–(−0.02))**	**0.021**
Methotrexate	−0.05 (−0.2–0.1)	0.55		
Azathioprine	0.08 (−0.02–0.2)	0.12	0.09 (−0.02–0.2)	0.099
Anti-TNF therapy	**0.1 (0.01–0.2)**	**0.027**	**0.1 (0.009–0.2)**	**0.033**
Ustekinumab	−0.2 (−0.5–0.02)	0.076	−0.2 (−0.4–0.08)	0.18
Vedolizumab	−0.1 (−0.4–0.2)	0.40		
Tofacitinib	1 (−0.2–0.4)	0.56		

In this analysis MDA (malondialdehyde) is the dependent variable. CRP: C reactive protein; TNF: tumor necrosis factor; CDAI: Crohn’s Disease Activity Index. Significant *p* values are depicted in bold.

**Table 3:** Cardiovascular disease data in relation to MDA serum levels.

		MDA, nmol/mL			
		Beta Coefficient (95% Confidence Interval), *p*			
		Univariable		Multivariable	
SCORE2, %	2.3 (1.0–4.4)	0.008 (−0.008–0.02)	0.33		
Low risk	140 (71)	-			
Moderate risk	49 (25)	0.02 (−0.09–0.1)	0.74		
High risk	7 (4)	0.1 (−0.1–0.4)	0.29		
Carotid assessment *					
Carotid plaque, n (%)	68 (35)	0.07 (−0.03–0.2)	0.19	0.03 (−0.07–0.1)	0.55
Bilateral, n (%)	35 (18)	**0.2 (0.03–0.3)**	**0.017**	0.1 (−0.03–0.2)	0.12
cIMT, mm	0.644 ± 0.137	0.3 (−0.09–0.6)	0.14	0.1 (−0.2–0.5)	0.47
Insulin resistance indices					
Glucose, mg/dL	95 ± 19	−0.001 (−0.003–0.001)	0.42		
Insulin, µU/mL	6.7 (4.8–11.1)	−0.001 (−0.008–0.006)	0.76		
C peptide, ng/mL	0.72 ± 0.43	0.009 (−0.1–0.1)	0.87		
HOMA2-IR	0.86 (0.62–1.46)	−0.007 (−0.06–0.04)	0.76		
HOMA2-S%	131 ± 82	−0.00001 (−0.00006–0.0006)	0.96		
HOMA2-B%	119 ± 49	0.003 (−0.0007–0.001)	0.53		
Lipid profile **					
Cholesterol, mg/dL	201 ± 49	**0.001 (0.0003–0.002)**	**0.012**	**0.001 (0.0002–0.002)**	**0.016**
Triglycerides, mg/dL	151 ± 89	**0.0006 (0.00004–0.001)**	**0.037**	0.0004 (−0.0001–0.001)	0.12
HDL, mg/dL	57 ± 18	−0.0002 (−0.002–0.003)	0.91		
LDL, mg/dL	116 ± 40	**0.001 (0.0001–0.003)**	**0.032**	**0.001 (0.0001–0.002)**	**0.049**
LDL:HDL ratio	2.18 ± 0.86	**0.06 (0.004–0.1)**	**0.035**	0.04 (−0.02–0.1)	0.17
Non-HDL, mg/dL	146 ± 43	**0.002 (0.0005–0.003)**	**0.004**	**0.001 (0.0003–0.003)**	**0.012**
Lipoprotein (a), mg/dL	26 (8–88)	**−0.0008 (−0.001–(−0.0002))**	**0.010**	**−0.0007 (−0.001–(−0.001))**	**0.022**
Apolipoprotein A1, mg/dL	162 ± 37	0.0003 (−0.001–0.002)	0.61		
Apolipoprotein B, mg/dL	108 ± 32	0.001 (0–0.003)	0.051	0.001 (−0.0005–0.003)	0.17
Apo B:Apo A1 ratio	0.69 ± 0.22	2 (−0.05–0.4)	0.13	0.07 (−0.2–0.3)	0.56
Atherogenic index	3.8 ± 1.2	**0.05 (0.009–0.09)**	**0.016**	0.03 (−0.009–0.08)	0.12
Apolipoprotein C-III, mg/dL	3.5 (2.8–4.4)	−0.008 (−0.03–0.01)	0.47		

In this analysis MDA (malondialdehyde) is the dependent variable. cIMT: Carotid intima media thickness; HDL: high-density lipoprotein; LDL: low-density lipoprotein; SCORE2: Systematic Coronary Risk Evaluation-2; HOMA2: homeostatic model assessment. * Adjusted for sex, BMI, hypertension and dyslipidemia. ** Adjusted for sex, BMI, and hypertension. Significant *p* values are depicted in bold.

**Table 4:** Liver disease in relation to MDA.

		MDA, nmol/mL			
		Beta Coefficient (95% Confidence Interval), *p*			
		Univariable		Multivariable	
FIB-4	0.93 ± 0.42	**2 (−0.05–0.3)**	**0.005**	**0.1 (0.03–0.3)**	**0.017**
Low risk	175 (89)	-			
Indeterminate	22 (11)	0.1 (−0.02–0.3)	0.092	0.1 (− 0.05–0.3)	0.18
High	0 (0)	-			
Fibroscan, kPa	0.97 ± 0.64	**0.02 (0.0008–0.04)**	**0.040**	0.01 (−0.006–0.03)	0.18
F0–F1	140 (86)	-		-	
F2	14 (9)				
F3	5 (3)	0.2 (0.05–0.3) *	**0.008**	**0.2 (0.03–0.3)**	**0.021**
F4	3 (2)				
Ultrasound grade of steatosis					
0	89 (51)	-		-	
1	53 (31)	0.1 (−0.01–0.2) **	**0.030**	0.09 (−0.02–0.2)	0.11
2 or 3	31 (18)				

In this analysis MDA (malondialdehyde) is the dependent variable. Multivariable analysis is adjusted for sex, BMI, hypertension, and dyslipidemia. FIB-4: Fibrosis-4 Index. * Refers to the beta coefficient when comparing F2 to F4 vs. F0:F1. ** Refers to the beta coefficient when comparing grades 1 to 3 vs. 0. Significant *p* values are depicted in bold.

## Data Availability

The data sets used and/or analyzed in the present study are available from the corresponding author upon request.
